# Hyperhomocysteinemia Increases Risk of Metabolic Syndrome and Cardiovascular Death in an Elderly Chinese Community Population of a 7-Year Follow-Up Study

**DOI:** 10.3389/fcvm.2021.811670

**Published:** 2022-02-10

**Authors:** Chang Liu, Liping Liu, Yinglu Wang, Xiaoli Chen, Jie Liu, Sheng Peng, Jingjiang Pi, Qi Zhang, Brain Tomlinson, Paul Chan, Lin Zhang, Huimin Fan, Liang Zheng, Zhongmin Liu, Yuzhen Zhang

**Affiliations:** ^1^Key Laboratory of Arrhythmias of the Ministry of Education of China, Research Center for Translational Medicine, Shanghai East Hospital, Tongji University School of Medicine, Shanghai, China; ^2^Department of Emergency Medicine, Shanghai East Hospital, Tongji University School of Medicine, Shanghai, China; ^3^Department of Cardiology, Shanghai East Hospital, Tongji University School of Medicine, Shanghai, China; ^4^Faculty of Medicine, Macau University of Science and Technology, Macau, China; ^5^Division of Cardiology, Department of Internal Medicine, Wan Fang Hospital, Taipei Medical University, Taipei, Taiwan

**Keywords:** hyperhomocysteinemia, abdominal obesity, metabolic syndrome, cardiovascular death, Chinese elderly population

## Abstract

**Background:**

Hyperhomocysteinemia (HHcy) and abdominal obesity are risk factors for metabolic syndrome (MetS) and death from cardiovascular disease (CVD). Recent studies have shown a correlation between HHcy and abdominal obesity, suggesting that they may have a combined effect on the risk of MetS and CVD mortality. However, this suspicion remains to be confirmed, particularly in the elderly population. We explored their combined effects on the risk of MetS and CVD mortality among the community population aged 65 and above in China.

**Methods and Results:**

This prospective study enrolled 3,675 Chinese community residents aged 65 and above in May 2013 with 7-year follow-up of all-cause and CVD mortality. HHcy was defined as the blood homocysteine (Hcy) level >15 μmol/L and abdominal obesity as waist circumference (WC) ≥90 cm for men and ≥80 cm for women (HWC). All participants were grouped into four categories by WC and the blood level of Hcy: NWC (normal WC) /HHcy(–), NWC/HHcy(+), HWC/HHcy(–), and HWC/HHcy(+). The relationship of combined HHcy and abdominal obesity with MetS and metabolic profile was evaluated by logistic regression analysis and the association of combined HHcy and abdominal obesity with CVD and all-cause mortality evaluated by Cox regression analysis. The prevalence of HHcy, abdominal obesity and MetS in elderly Chinese community residents was 40.1, 59.3, and 41.4%, respectively. Using group without HHcy and abdominal obesity [NWC/HHcy(–)] as reference, the participants of other three groups had significantly higher risk of MetS and its component abnormalities, with HWC/HHcy(+) group having the highest risk (OR = 13.52; 95% CI = 8.61–14.55). After a median of 6.94 (±1.48) years follow-up, 454 deaths occurred with 135 CVD deaths. Compared with NWC/HHcy(–) group, the risk of 7-year follow-up CVD mortality (HR = 1.75; 95% CI = 1.02–3.03) and all-cause mortality (HR = 1.23; 95% CI = 1.04–2.18) of HWC/HHcy(+) group increased considerably after adjustment for major MetS and CVD risk factors.

**Conclusions:**

There is high prevalence of HHcy, abdominal obesity, and MetS in the elderly Chinese community population. HHcy increases risk of MetS, CVD, and all-cause mortality, especially in the populations with abdominal obesity.

## Introduction

China is the most populous country on Earth and the population aging in China is serious. According to United Nations, the proportion of people aged 65 and over in China will reach 26.1% around 2050, which exceeds 10% of average population aging globally (1). Cardiovascular disease (CVD) is one of the leading causes of death worldwide, especially in the elderly population. Report on Cardiovascular Health and Diseases in China 2019 showed that there still had been an increase of CVD prevalence, with a projected current prevalence of around 330,000,000, and the first rank CVD mortality has imposed a heavy burden in many healthcare systems.

Metabolic syndrome (MetS) is a cluster of conditions that occur together, and it significantly increases CVD mortality (2, 3). The conditions of MetS include high blood pressure, high blood sugar, excess body fat around the waist, and abnormal blood cholesterol or triglyceride levels. MetS is increasingly common, with 25–35% of the adult population having MetS (4, 5). Over the past few decades, researchers tried to identify the risk factors contributing to raise the risk of MetS or related CVD mortality, although the independent risk factor has failed to deliver the desirable effects in prediction or control of disease or mortality risk. Due to the complexity of human body and its surroundings, diseases usually are not triggered by a single factor. Therefore, we should pay more attention to the combined effects of different risk factors.

Homocysteine (Hcy) is a sulfur amino acid and the byproduct in the conversion of methionine to cysteine. Blood Hcy levels are determined by several factors, such as the cofactors vitamin B12, vitamin B6, and folic acid and enzymes involved in methionine metabolism. Serum blood Hcy levels >15 μmol/L is defined as Hyperhomocysteinemia (HHcy) (6). After its discovery, Hcy was considered as a risk factor for development of atherosclerotic plaques in blood vessels leading to occurrence of CVD.

In 1976, Wilcken and Wilcken (7) first provided evidence for an association between elevated Hcy levels and coronary artery disease in general population. Further, a meta-analysis of 29 epidemiologic studies provided considerable evidence that elevated Hcy levels were associated with an increased risk of arteriosclerotic vascular disease (8). Since then, a growing number of studies confirmed the association of HHcy with CVD or CVD mortality (9–13). A recent large trial of Hcy reduction in hypertensive patients without a history of stroke or myocardial infarction in China resulted in a 21% reduction of stroke risk, suggesting that homocysteine reduction could be considered as primary prevention for CVD (14). In addition to CVD, HHcy has been found to be related to other diseases, such as neurodegenerative disease, stroke (15), and small vessel disease (16).

Abdominal obesity has been closely related to a variety of metabolic disorders such as type 2 diabetes, cardiovascular diseases, and total mortality (17, 18). Recent studies reported a correlation between abdominal obesity and HHcy, and changes in body composition especially abdominal obesity predicting HHcy (19–21). These studies suggest a possibility that abdominal obesity and HHcy may have the synergistic effects on the risk of MetS and CVD mortality.

Many previous studies provided important information of HHcy and abdominal adiposity, but the studies usually were in the general population. However, the fat distribution and blood Hcy level change with increasing age (22, 23), and less study was reported in an elderly population and no study of the combined effects of HHcy and abdominal obesity on the risk of MetS and CVD mortality.

Therefore, in this study we used our previous recruited community residents of Shanghai Elderly Cardiovascular Health Study (SHECHS) (24) to investigate the prevalence of HHcy, abdominal obesity in this Chinese elderly population. With a 7-year follow up for all-cause and CVD mortality of the population, we further explore the impact of combine effects of HHcy and abdominal obesity on the risk of MetS and CVD mortality.

## Materials and Methods

### Study Design and Population

The Shanghai Elderly Cardiovascular Health Study (SHECHS) is a community population-based longitudinal study of non-institutionalized older individuals in Gaohang community (24). All people age ≥65 years who were permanent residents in the community were invited to participate in the study. Baseline information and clinical characteristics were collected through questionnaires, physical examinations and laboratory tests, and all-cause and CVD mortality were collected after a 7-year follow-up. The validation of the sued study questionnaire was performed. A total of 3,950 subjects were enrolled in May 2013. The exclusion criteria were: (1)missing values of blood Hcy or waist circumference (WC); (2)severe renal insufficiency as glomerular filtration rate (GFR) <60 ml/min/1.73 m^2^ calculated with a modified version of the Modification of Diet in Renal Disease (MDRD) equation as follows: 175 × serum creatinine (mg/dL)−1.234 × age (year)−0.179 ×0.79 (if female) based on the Chinese population (25), thus 275 participants were excluded and the remaining 3,675 participants were included in this study. This study was conducted according to the principles established in the Declaration of Helsinki of 1975 and approved by institutional review board and ethics committee of Shanghai East Hospital, Tongji University of Medicine, and written informed consent was obtained from each participant before data collection.

### Demographic, Anthropometric, and Laboratory Measurements

The participants attending Gaohang community medical center were interviewed face-to-face by trained study staff to complete questionnaires covering sociodemographic information, medical history, and all current medications. WC was measured on standing position in the midway between the lower edge of the costal arch and the upper edge of the iliac crest using a non-elastic tape (to the nearest 0.5 cm). Body mass index (BMI) was calculated as weight in kilograms divided by height in meters squared. Height and weight were measured to the nearest 0.5 cm and 0.1 kg, respectively, with the participants wearing lightweight clothing and no shoes. All anthropometric measurements were taken in duplicate, and the averages of these measurements were used in the analyses. For blood pressure (BP), resting systolic BP (SBP), and diastolic BP (DBP) levels were measured three times at 1-min intervals using a standard mercury sphygmomanometer after a 5-min rest upon arrival. The average of the second and third readings was used in the analyses. Fasting venous blood samples were collected in the morning after at least 10 h of fasting. Blood samples were obtained on their arrival at the center and sent to the Blood Laboratory of Tongji Medical School affiliated Shanghai East Hospital for processing and measurement within 2 h. Fasting serum glucose, lipid profile, and uric acid were measured enzymatically on the Roche Cobas 8000 C702 Biochemistry system. HbA1c was measured by ion-exchange high-performance liquid chromatography on the ToSoH G8 analyzer and hsCRP was measured by particle enhanced immunonephelometry. Serum total (free plus protein bound) Hcy was measured with tri-n-butylphosphine as the reducing agent and ammonium 7-fluorobenzo-2-oxa-1,3-diazole-4-sulfonate as the thiol-specific fluorochromophore, followed by high-performance liquid chromatography with fluorescence detection (26).

### Definition

HHcy was diagnosed as serum blood Hcy levels >15 μmol/L (6).

MetS was defined based on the International Diabetes Federation (IDF) Scientific Statements (27). Diagnosis of the syndrome was established when at least three of the following five components were present:

1. WC: elevated waist circumference (abdominal obesity) > 90 cm in men and > 80 cm in women;
2. TG disorder: serum triglycerides levels ≥1.70 mmol/L or taking lipid-lowering drugs;
3. HDL disorder: reduced HDL-C, <1.03 mmol/L in men or <1.29 mmol/L in women;
4. Hypertension: elevated blood pressure, systolic ≥130 and/or diastolic ≥85 mm Hg (1 mmHg = 0.133 kPa) or taking anti-hypertensive drugs;
5. Diabetes: elevated fasting glucose ≥5.56 mmol/L or taking hypoglycemic drugs.

### Statistical Analysis

The basic characteristics of the study population are summarized as mean (standard deviation, SD) for continuous variables and as percentages for categorical variables. One-way analysis of variance (ANOVA) along with Kruskal–Wallis test was used to compare quantitative variables between groups; the distribution of the categorical variables was tested by χ^2^ analysis. Binary and multinomial logistic regression models were used to obtain the ORs with 95% CI as estimates of the associations between combined effect of HHcy and abdominal obesity and MetS components. Then, we compared the CVD and all-cause death survival curve using Kaplan–Meier analysis and the log-rank test after dividing the populations with abdominal obesity into a HHcy and a non-HHcy cohort. Cox regression analysis was used to determine the relationship between HHcy and all-cause and CVD death. Estimates were adjusted for two models (Model 1: age; Model 2: age, sex, education, smoking, alcohol drinking, SBP, DBP, hypoglycemic therapy, lipid-lowering therapy, and creatinine). All data were analyzed using SPSS Statistics software version 25.0 for Macintosh (IBM Corp., Armonk, New York), and the significance level for all analyses was set at *P* < 0.05.

## Results

### Demographic and Clinical Characteristics of Study Subjects

The baseline characteristics of the participants were presented in Table 1. Among 3,675 participants, 44.0% was male and the mean age was 72. There was no significant difference of age between men and women. 66.3% of participants had an average monthly income of <2,500, and the majority (89.6%) had less than a lower secondary education (i.e., middle school educated or less). Compared with males, female participants had fewer current smokers and drinkers. More men than women were self-reported as being physically active with 91.2% of men regarded as regularly compared to 89.0% of women (*P* = 0.012). In the whole cohort, the prevalence of MetS was 41.4%, and men had significantly lower prevalence of MetS than women (31.2% in men vs. 49.4% in women, *P* < 0.001). The mean Hcy was 15.9 μmol/L, with a higher level in men than in women (17.9 μmol/L in men vs. 14.3 μmol/L in women, *P* < 0.001). The prevalence of HHcy was 40.1%, with 53.6% in men and 29.4% in women (*P* < 0.001). Women had a little higher BMI than men (24.7 kg/m^2^ in women vs. 24.5 in men, *p* = 0.012), however, significantly higher prevalence of abdominal obesity was observed in women (70.3%) than in men (45.3%; *P* < 0.001; Table 1).

**Table 1:** Baseline characteristics of study population.

	**Total (*n* = 3,675)**	**Male (*n* = 1,617, 44.0%)**	**Female (*n* = 2,058, 56.0%)**	***P*-value**
Age, years^*^	72.0 (6.6)	71.8 (6.5)	72.1 (6.7)	0.098
BMI, kg/m^2^*^^	24.6 (3.4)	24.5 (3.1)	24.7 (3.6)	0.012
WC, cm^*^	86.7 (9.4)	88.1 (9.4)	85.6 (9.3)	<0.001
HWC, n (%)	2,179 (59.3%)	733(45.3%)	1,446 (70.3%)	<0.001
**Average monthly income, CNY**, ***n*** **(%)**
≤ 2,500	2,621 (66.3%)	977 (56.7%)	1,644 (75.4%)	<0.001
>2,500	1,283 (32.5%)	74643.3%	537 (24.6%)	<0.001
**Education, years**, ***n*** **(%)**
≤ 12	3,542 (89.6%)	1443 (82.8%)	2,099 (95.4%)	<0.001
>12	402 (10.2%)	300 (17.2%)	102 (4.6%)	<0.001
**Lifestyle**, ***n*** **(%)**
Current smoking	556 (14.1%)	534 (40.6%)	22 (1.0%)	<0.001
Current drinking	542 (13.7%)	512 (29.3%)	30 (1.3%)	<0.001
Physical activity	3 559 (90.1%)	1599 (91.2%)	1960 (89.0%)	0.012
MetS	1 522 (41.4%)	505 (31.2%)	1017 (49.4%)	<0.001
MetS components^*^
SBP, mmHg	138.8 (17.3)	138.5 (17.4)	139.1 (17.2)	0.224
DBP, mmHg	81.9 (8.9)	82.5 (9.2)	81.3 (8.6)	<0.001
FG, mmol/L	5.76 (1.87)	5.68 (1.85)	5.82 (1.88)	0.018
TC, mmol/L	4.99 (0.97)	4.72 (0.91)	5.20 (0.97)	<0.001
TG, mmol/L	1.63 (1.16)	1.53 (1.08)	1.71 (1.22)	<0.001
HDL-C, mmol/L	1.47 (0.40)	1.39 (0.39)	1.53 (0.40)	<0.001
LDL-C, mmol/L	3.31 (0.88)	3.12 (0.84)	3.46 (0.88)	<0.001
Hcy, μmol/L^*^	15.9 (9.2)	17.9 (10.8)	14.3 (7.2)	<0.001
HHcy, *n* (%)	1,473 (40.1%)	867 (53.6%)	606 (29.4%)	<0.001

*Data shown as mean (SD)*

* * for continuous variables and n (%) for categorical variables*.

*P-values comparing men and women*.

*WC, waist circumference; BMI, body mass index; MetS, metabolic syndrome; SBP, systolic blood pressure; DBP, diastolic blood pressure; FG, fast plasma glucose; TC, total cholesterol; TG, triglycerides; HDL–C, high–density lipoprotein cholesterol; LDL-C, low–density lipoprotein cholesterol; Hcy, serum total homocysteine; HHcy, hyperhomocysteinamia; HWC, higher WC (≥90 cm for men and ≥80 cm for women)*.

### The Changes of Metabolic Profile Groups According to WC and Blood Homocysteine Level

Less study was reported in elderly population of HHcy and abdominal obesity on MetS and CVD mortality, thus, we divided the participants into four categories according to WC and blood Hcy level: NWC/HHcy (–), NWC/HHcy (+), HWC/HHcy (–), and HWC/HHcy (+) and analyzed the changes of metabolic profiles in different groups (Table 2). NWC is defined as a normal WC (<90 cm for men and <80 cm for women), and HWC is defined as higher WC (≥90 cm for men and ≥80 cm for women) (27); HHcy (+) and HHcy (–) represented the individuals with or without HHcy (6) (Table 2). The HWC/HHcy (–) group (35.5%) was the most prevalent among the participants, followed by NWC/HHcy (–) (24.4%), HWC/HHcy (–) (23.8%), and NWC/HHcy (+) (16.3%). There was a significantly increased age in HHcy group of normal WC (73.4 vs. 69.8 years, *P* < 0.05) and HHcy group of high WC group (74.8 vs. 70.8 years, *P* < 0.05) compared to the non-HHcy group, with the most significant age increase of HWC/HHcy (+) in comparison to the other three groups. In group of NWC/HHcy (–), NWC/HHcy (+), HWC/HHcy (–), and HWC/HHcy (+), there was an increase of SBP with significance only in HWC/HHcy (+) group [141.9 mmHg vs. 135.4, 137.0, and 139.7 mmHg in NWC/HHcy (–), NWC/HHcy (+), HWC/HHcy (–), respectively], and a significant reduction of HDL-C in HWC/HHcy (–) (1.43 mmol/L) and HWC/HHcy (+) (1.38 mmol/L) compared to NWC/HHcy (–), NWC/HHcy (+) group with no significance observed in the normal WC group [1.59 and 1.48 mmol/L in NWC/HHcy (–), NWC/HHcy (+) group]. A significant increase of FG and TG was also observed in HWC/HHcy (–) and HWC/HHcy (+) group compared to NWC/HHcy (–), NWC/HHcy (+) group (Table 2). These results suggest that significant changes of metabolic profile in HHcy group especially with HWC.

**Table 2:** Metabolic profile of the study group categorized by WC and Hcy.

	**NWC/HHcy (–)**	**NWC/HHcy (+)**	**HWC/HHcy (–)**	**HWC/HHcy (+)**
	**(*n* = 897, 24.4%)**	**(*n* = 599, 16.3%)**	**(*n* = 1,305, 35.5%)**	**(*n* = 874, 23.8%)**
Age, years^*^	69.8 (5.4)	73.4 (6.9)^a^	70.8 (5.9)^a^^,^^b^	74.8 (6.9)^a^^−^^c^
WC, cm^*^	78.3 (6.9)	80.0 (6.3)	91.0 (7.2)^a^^,^^b^	93.5 (7.5)^a^^,^^b^
BMI, kg/m^2^*^^	22.2 (2.5)	22.4 (2.5)^a^	26.2 (3.1)^a^^,^^b^	26.2 (3.1)^a^^,^^b^
TG, mmol/L^*^	1.39 (0.98)	1.42 (0.96)^a^	1.79 (1.31)^a^^,^^b^	1.76 (1.09)^a^^,^^b^
TC, mmol/L^*^	5.02 (0.95)	4.84 (0.96)^a^	5.08 (1.00)	5.00 (0.97)^c^
HDL-C, mmol/L^*^	1.59 (0.43)	1.48 (0.42)	1.43 (0.35)^a^^,^^b^	1.38 (0.37)^a^^,^^b^
LDL-C, mmol/L^*^	3.30 (0.86)	3.16 (0.87)^a^	3.41 (0.89)^a^	3.30 (0.87)^c^
SBP, mmHg^*^	135.4 (16.9)	137.0 (18.0)	139.7 (16.9)	141.9 (16.8)^b^
DBP, mmHg^*^	80.5 (8.6)	81.2 (9.2)	82.4 (8.6)	82.3 (9.1)^a^^,^^b^
FG, mmol/L^*^	5.51 (1.73)	5.34 (1.63)^a^	6.06 (1.97)^a^^,^^b^	5.93 (2.01)^a^^,^^b^
HbA1c,%	6.25 (1.05)	6.20 (1.08)	6.55 (1.15)^a^^,^^b^	6.39 (1.09)^a^^−^^c^
**Metabolic profile**, ***n*** **(%)**
High FG	250 (27.9%)	141 (23.5%)	575 (44.1%)	348 (39.8%)
High TG	193 (21.5%)	133 (22.2%)	528 (40.5%)	345 (39.5%)
Low HDL	119 (13.3%)	93 (15.5%)	377 (28.9%)	241 (27.6%)
High BP	634 (70.7%)	449 (75.0%)	1,044 (80.0%)	745 (85.2%)
MetS	100 (11.1%)	76 (12.7%)	815 (62.5%)	531 (60.8%)

*Data shown as mean (SD)*

* * for continuous variables and n (%) for categorical variables*.

*NWC: normal WC (<90 cm for men and <80 cm for women); HWC, higher WC (≥90 cm for men and ≥80 cm for women); HHcy (–), without HHcy; HHcy (+), with HHcy*.

*WC, waist circumference; HHcy, hyperhomocysteinamia; BMI, body mass index; TG, triglycerides; TC, total cholesterol; HDL–C, high–density lipoprotein cholesterol; LDL-C, low–density lipoprotein cholesterol; SBP, systolic blood pressure; DBP, diastolic blood pressure; FG, fast plasma glucose; MetS: metabolic syndrome*.

a *P <0.05, compared with NWC/HHcy(–)*.

b *P <0.05, compared with NWC/HHcy(+)*.

c *P <0.05, compared with HWC/HHcy(–)*.

### Increase MetS Risk With Highest in HWC/HHcy (+) Group

Using subjects of NWC/HHcy (–) as the reference group, the subjects in groups NWC/HHcy (+), HWC/HHcy (–), HWC/HHcy (+) had increased risk of MetS with OR = 1.47 (95% CI = 1.04–2.09), OR = 13.48 (95% CI = 10.37–17.52) and OR = 15.02 (95% CI = 11.28–20.00), respectively, especially HWC/HHcy (+) group having the highest risk of metabolic disorders. Whereas, in comparison of the abnormality of MetS components, the participants in HWC/HHcy (+) group had the highest risk of TG disorder (OR = 2.74; 95% CI = 2.17–3.47), HDL disorder (OR = 2.44; 95% CI = 1.85–3.23), and Hypertension (OR = 2.33; 95% CI = 1.79–3.05) after adjusting for age, sex, current smoking, current drinking, activity, hypoglycemia therapy, lipid-lowering therapy, and creatinine. However, the risk of blood glucose disorders was slightly lower in participants with both HHcy and high WC than in participants without HHcy or high WC [(OR = 1.92; 95% CI = 1.51–2.45) vs. (OR = 2.01; 95% CI = 1.62–2.49; Table 3).

**Table 3:** ORs of MetS and the component abnormality among the study population.

		**NWC/HHcy (–)**	**NWC/HHcy (+)**	**HWC/HHcy (–)**	**HWC/HHcy (+)**
		**(*n* = 897, 24.4%)**	**(*n* = 599, 16.3%)**	**(*n* = 1,305, 35.5%)**	**(*n* = 874, 23.8%)**
High FG	Model 1	1.00	0.80 (0.63, 1.01)	2.04 (1.70, 2.45)	1.71 (1.40, 2.09)
	Model 2	1.00	0.89 (0.72, 1.10)	2.20 (1.85, 2.61)	1.73 (1.42, 2.10)
	Model 3	1.00	088 (0.66, 1.16)	2.01 (1.62, 2.49)	1.92 (1.51, 2.45)
High TG	Model 1	1.00	1.04 (0.81, 1.34)	2.48 (2.04, 3.01)	2.38 (1.93, 2.93)
	Model 2	1.00	0.95 (0.77, 1.17)	1.97 (1.66, 2.34)	2.00 (1.65, 2.42)
	Model 3	1.00	1.32 (1.01, 1.74)	2.50 (2.02, 3.09)	2.74 (2.17, 3.47)
Low HDL—C	Model 1	1.00	1.20 (0.90, 1.61)	2.66 (2.12, 3.33)	2.49 (1.95, 3.18)
	Model 2	1.00	1.18 (0.93, 1.51)	2.05 (1.68, 2.49)	2.05 (1.64, 2.55)
	Model 3	1.00	1.64 (1.18, 2.27)	2.14 (1.67, 2.74)	2.44 (1.85, 3.23)
High BP	Model 1	1.00	1.24 (0.98, 1.57)	1.66 (1.36, 2.02)	2.40 (1.89, 3.03)
	Model 2	1.00	1.27 (1.03, 1.56)	2.06 (1.70, 2.50)	3.39 (2.62, 4.38)
	Model 3	1.00	1.09 (0.84, 1.41)	1.61 (1.30, 2.00)	2.33 (1.79, 3.05)
MetS	Model 1	1.00	1.11 (0.86, 1.43)	11.52 (9.43, 14.06)	10.47 (8.41, 13.03)
	Model 2	1.00	1.16 (0.84, 1.59)	13.26 (10.47, 16.79)	12.34 (9.63, 15.82)
	Model 3	1.00	1.47 (1.04, 2.09)	13.48 (10.37, 17.52)	15.02 (11.28, 20.00)

*NWC, normal WC (<90 cm for men and <80 cm for women); HWC, higher WC (≥90 cm for men and ≥80 cm for women); HHcy (–): without HHcy; HHcy (+): with HHcy*.

NWC/HHcy (–) group used as reference. ^*^P <0.05.

*Model 1: Unadjusted*.

*Model 2: Data adjusted for age*.

*Model 3: Data adjusted for age, sex, current smoking, current drinking, activity, hypolycemia therapy, lipid-lowering therapy, and creatinine*.

*WC, waist circumference; HHcy, hyperhomocysteinamia; FG, fast plasma glucose; TG, triglycerides; HDL, high–density lipoprotein cholesterol; BP, blood pressure; MetS: metabolic syndrome; OR, odds ratio*.

### Increase the Risk of CVD and All-Cause Mortality With Highest in HWC/HHcy (+) Group

**Figure 1:**
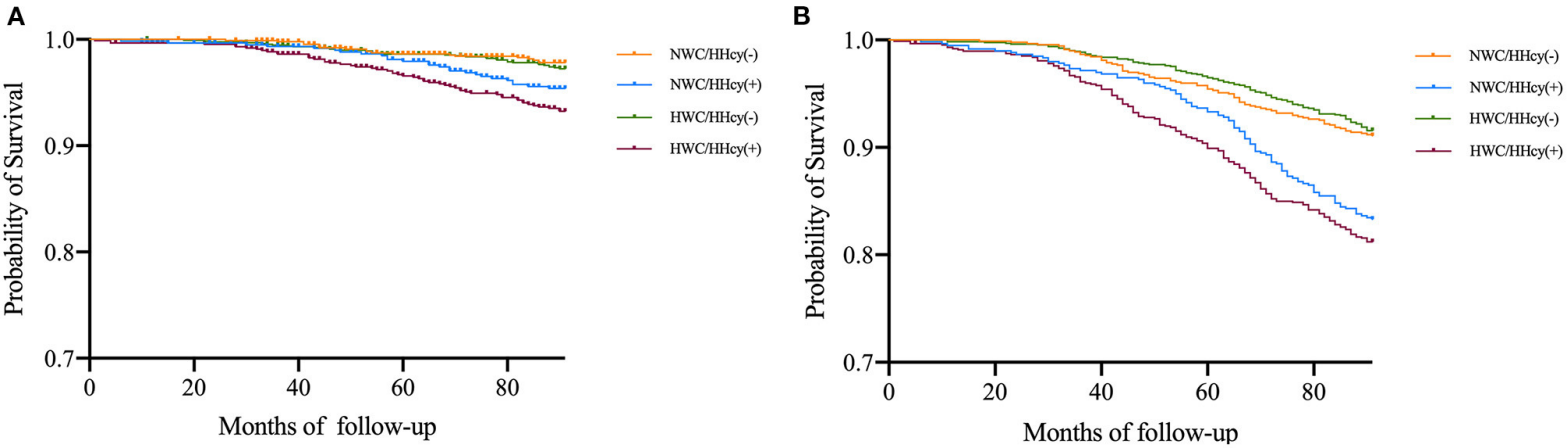
**(A)** Estimated survival among WC/HHcy groups with Kaplan–Meier survival analysis. The figure shows the survival estimated with Kaplan-Meier product-limit method in cohort grouped by WC/HHcy. Ending event is cardiovascular death. NWC represents normal WC (<90 cm for men and <80 cm for women); HWC represents higher WC (≥90 cm for men and ≥80 cm for women); HHcy (–) represents without HHcy; HHcy (+) represents with HHcy. **(B)** Estimated survival among WC/HHcy groups with Kaplan–Meier survival analysis. The figure shows the survival estimated with Kaplan–Meier product-limit method in cohort grouped by WC/HHcy. Ending event is all-cause death. NWC represents normal WC (<90 cm for men and <80 cm for women); HWC represents higher WC (≥90 cm for men and ≥80 cm for women); HHcy (–) represents without HHcy; HHcy (+) represents with HHcy.

After follow-up of a median of 6.94 (±1.48) years, 454 deaths occurred among 3,675 participants, of whom 135 died of CVD. The 319 non-cardiovascular deaths were mainly due to malignant neoplasms, septicemia, and other causes. In the entire cohort, the 7-year mortality of CVD was 3.7% in whole population, 3.0% in the participants with abdominal obesity, and 4.1% in the participants with HHcy; and all-cause mortality was 12.4, 12.0, and 12.6%, respectively. Among the four groups, the HWC/HHcy (+) group had the highest CVD mortality, followed by NWC/HHcy (+) group, and similar results were observed in all-cause mortality (Figure 1 and Table 4).

**Table 4:** HRs for 7-year CVD and all-cause death associated with WC/HHcy groups.

	**NWC/HHcy (–)**	**NWC/HHcy (+)**	**HWC/HHcy (–)**	**HWC/HHcy (+)**
	**(*n* = 897, 24.4%)**	**(*n* = 599, 16.3%)**	**(*n* = 1,305, 35.5%)**	**(*n* = 874, 23.8%)**
**CVD death**
Case	19	26	35	55
Mortality	2.1%	4.3%	2.7%	6.3%
Model 1	1.00	2.12 (1.17, 3.83)^*^	1.26 (0.72, 2.19)	3.16 (1.87, 5.32)^*^
Model 2	1.00	1.34 (0.73,2.46)	1.13 (0.64, 2.00)	1.79 (1.05, 3.07)^*^
Model 3	1.00	1.36 (0.74, 22.51)	1.06 (0.60, 1.89)	1.75 (1.02, 3.03)^*^
**All-cause death**
Case	80	100	110	164
Mortality	8.9%	16.7%	8.4%	18.7%
Model 1	1.00	1.74 (1.24, 2.50)^*^	0.94 (0.70, 1.25)	2.24 (1.71,2.92)^*^
Model 2	1.00	1.23 (1.02,1.67)^*^	0.90 (0.67,1.21)	1.36 (1.18,2.20)^*^
Model 3	1.00	1.16 (0.94,1.60)	0.82 (0.60,1.12)	1.23 (1.04,2.18)^*^

*NWC, normal WC (<90 cm for men and <80 cm for women); HWC, higher WC (≥90 cm for men and ≥80 cm for women); HHcy (–): without HHcy; HHcy (+): with HHcy*.

*NWC/HHcy (–) group used as reference*.

* *P <0.05*.

*Model 1: Unadjusted*.

*Model 2: Data adjusted for age, sex, current smoking, current drinking and activity*.

*Model 3: Data adjusted for age, sex, current smoking, current drinking, activity, hypolycemia therapy, lipid-lowering therapy and creatinie*.

*WC, waist circumference; HHcy, hyperhomocysteinamia; CVD, cardiovascular disease; HR, hazard ratio*.

Compared with the NWC/HHcy (–) group, the risk of 7-year CVD mortality and all-cause mortality increased considerably in other three groups. Adjustment for age, sex, current smoking, current drinking, activity, hypoglycemia therapy, lipid-lowering therapy, and creatinine did not change the association of abdominal obesity and HHcy combination with CVD mortality [(HR = 1.75; 95% CI = 1.02–3.03)], and all-cause mortality [(HR = 1.23; 95% CI = 1.04–2.18)]. However, no significant association was observed in both CVD and all-cause mortality with NWC/ HHcy (+) and HWC/HHcy (–) groups (Table 4). These results suggest that HWC/HHcy(+) group has the highest risk of CVD and all-cause mortality.

## Discussion

In this prospective, population-based follow-up study of 3,675 participants aged 65 and over in the community of Shanghai China, we observed a high prevalence of HHcy, abdominal obesity and MetS in this elderly population, and HHcy increases the risk of MetS. Moreover, with 7-year-follow-up study of the elderly population, we found HHcy is a risk factor for CVD mortality and all-cause mortality that appears stronger in abdominal obese individuals than in non-abdominal obese individuals.

We observed that the prevalence of HHcy was 40.1%, with a higher Hcy level in men than in women (17.9 μmol/L in men vs. 14.3 μmol/L in women). The Hcy level and the prevalence of HHcy were higher than that reported in Netherland (14.3 μmol/L) (28), Norway (12.9 μmol/L in males aged 65–67 and 11.6 μmol/L in females aged 65–67) (29) and the US (11.5 μmol/L in males aged 60–69 and 10.3 μmol/L in females aged 60–69; 13.0 μmol/L in males aged 70–79 and 11.4 μmol/L in females aged 70–79; 13.6 μmol/L in males aged 80 and 12.6 μmol/L in females aged 80) (30). Serum Hcy level and the prevalence of HHcy reported in different studies vary considerably, and these differences reported by countries may be due to different population inclusion criteria, genetic background, and differences in the profile of HHcy risk factors (e.g., nutritional status and lifestyle). It was reported that the incidence of HHcy was reduced in some developed countries due to policies of fortifying cereals and flour with folic acid. For example, before folic acid fortification, the prevalence of HHcy in North America was 18.7% in whole population (30), however, after fortification, the prevalence decreased to 9.8%. The HHcy prevalence and Hcy level in China is higher than that in many developed countries, especially in the elderly, probably due to less intake of folic acid in cereals and flour. The high prevalence of HHcy and its association with metabolic disorders are adverse events a matter of some concern. Although, elevated blood Hcy and MetS are associated with CVD and CVD mortality (31–34), but the association between Hcy and MetS is not well-characterized especially in the elderly population. MetS is a cluster of conditions that occur together, and it significantly increases CVD mortality (2, 3). Our study found that HHcy increased the overall risk of MetS in the elderly population, moreover, we demonstrated a positive association between HHcy and MetS.

In addition to the finding that HHcy increases the overall risk of MetS, our further analysis of the correlation between HHcy and various components of MetS showed that abnormal accumulation of the abdominal fat and reduction of HDL cholesterol level were important risk factors associated with HHcy. These results were in consistent with a previous Chinese centenarians study by Fu et al. (35). Apart from WC and HDL-C, they also found an association of HHcy with triglyceride. Therefore, our study adds more evidence of correlation between HHcy and MetS into the literatures.

Several possible mechanisms might be relevant to the association of HHcy and MetS. According to previous studies, the most important feature of the MetS is insulin resistance, and HHcy could be either the cause (36) or result (37) of insulin resistance, while insulin resistance might play an important role in the regulation of serum Hcy level (38). Cysteine-b-synthase, the key enzyme in the transsulfuration pathway of homocysteine metabolism, was reported downregulated in insulin-resistant states (39). Meanwhile, elevated serum homocysteine levels exacerbated insulin resistance by inhibiting insulin receptor kinase activity (40). *In vitro* cell culture and *in vivo* animal studies have also shown that insulin-resistant states may reduce rather than increase circulating Hcy levels due to enhanced Hcy catabolism (41, 42). In addition, HHcy was reported preceding the development of metabolic syndrome features in a fructose-fed animal model (43). This study used a relative large clinical elderly population and confirmed the association between HHcy and MetS, therefore, high Hcy level could possibly be an early marker or MetS.

With 7-year follow-up study of the elderly population, we observed HHcy was a risk factor for CVD mortality and all-cause mortality that occurred stronger in abdominal obese individuals than in non-abdominal obese individuals. This finding was consistent with several prospective studies that had investigated the relation between Hcy and risk of mortality. In a meta-analysis included 12 prospective studies, Peng et al. (44) indicated that elevated Hcy level was an independent predictor for subsequent CVD mortality or all-cause mortality, and there was a significant dose-response association between Hcy level and risk of CVD mortality and all-cause mortality. The pooled RR of CVD mortality and all-cause mortality for the per 5 μmol/L increment Hcy level was 1.32 and 1.27. Our study confirmed that HHcy increased both CVD and overall mortality in the elderly, especially in abdominal obese individuals, and the correlation between HHcy and CVD or overall mortality still existed after adjusted with MetS or CVD risk factors. In addition, no study was reported to look at the correlation of combined HHcy and abdominal obesity with CVD or overall mortality.

There was no study reported to look at the correlation of combined HHcy and abdominal obesity with CVD or overall mortality. The present study provides such an opportunity by using abdominal circumference as a measure of abdominal obesity to group the populations of different blood Hcy level. The stronger association between HHcy and mortality was observed among those with abdominal obesity than among those without abdominal obesity. This correlation can be explained from two mutually complementary perspectives. On one hand, high blood Hcy levels may be a consequence of abdominal obesity that alters the metabolic kinetics of homocysteine; on the other hand, high Hcy levels may be responsible for abdominal obesity through an altered pattern of epigenetic modifications. Blood homocysteine concentration was reported to be associated with an increase in the percentage of adipose tissue (21), which regulates body fat through the epigenetic expression of genes that contribute to the development and progression of obesity (45).

The current study has limitations. (1) WC can only provide a rough estimate of abdominal fat accumulation, and more accurate measurement of abdominal obesity and other ectopic adipose tissue by computed tomography (CT) and magnetic resonance imaging (MRI) (46) could be performed to further evaluate the combined effects of HHcy and ectopic fat accumulation with MetS and CVD mortality. (2) We do not have the data on the intake and serum levels of folic acid, vitamin B12, and vitamin B6, which plays an important role in the metabolism of homocysteine (47). Therefore, we were unable to further compare whether the relationship between B vitamins and serum homocysteine levels differed in influencing CVD and all-cause mortality in people with and without abdominal obesity. (3) We have only examined the association between blood Hcy and the risk of MetS and further CVD or overall mortality in follow-up study. However, parallel changes in various homocysteine metabolites occur during HHcy, so it is difficult to attribute the observed pathology to a specific metabolite. For example, there is growing evidence that the Hcy-thiolactone pathway leading to N-Hcy-protein (48) plays an important role in providing a mechanistic explanation for developing MetS and causing CVD death. (4) The study is only one center of Shanghai China community elderly population, and multiple center large population required to further confirm the results.

In conclusion, a high prevalence of HHcy, abdominal obesity and MetS is observed in Chinese Shanghai community elderly population. HHcy is a risk factor for cardiovascular mortality and overall mortality during 7-year follow-up, which showed a stronger association in abdominal obesity individuals. These findings suggest that the elderly population should have regular physical examinations and pay more attention to waist circumference and blood Hcy levels. Prompt intervention for abdominal obesity and HHcy may be effective in reducing the risk of MetS and CVD mortality.

## Data Availability Statement

The original contributions presented in the study are included in the article/Supplementary Materials, further inquiries can be directed to the corresponding author/s.

## Ethics Statement

The studies involving human participants were reviewed and approved by Institutional Review Board and Ethics Committee of Shanghai East Hospital, Tongji University of Medicine. The patients/participants provided their written informed consent to participate in this study.

## Author Contributions

CL, LL, YW, XC, and SP contributed to the acquisition of data, analysis and interpretation of data, and drafting of the article. LZha assisted with the formal methodology and analysis. JL and YZ assisted with the revision of important intellectual content in the article. HF, LZhe, and YZ were responsible for resources, supervision, and project management. All authors contributed to the article and approved the submitted version.

## Funding

This study was supported by the National Natural Science Foundation of China (81970233, 81970234, 81970232, 81903174 and 82130016); the Science and technology commission of Shanghai Municipality (19JC1414500); the Shanghai Rising-Star Program (20QA1408100) and the Peak Disciplines (Type IV) of Institutions of Higher Learning in Shanghai.

## Conflict of Interest

The authors declare that the research was conducted in the absence of any commercial or financial relationships that could be construed as a potential conflict of interest.

## Publisher's Note

All claims expressed in this article are solely those of the authors and do not necessarily represent those of their affiliated organizations, or those of the publisher, the editors and the reviewers. Any product that may be evaluated in this article, or claim that may be made by its manufacturer, is not guaranteed or endorsed by the publisher.

## Supplementary Material

Supplementary Figure 1 The representative figure of homocysteine metabolism in human. AdoMet, adenosylmethionine; MAT, methionine *S*-adenosyltransferase; GNMT, glycine N-methyltransferase; AdoHcy, S-Adenosyl-L-homocysteine; THF, tetrahydrofolate; MTHFR, methylenetetrahydrofolate reductase; MS, MS, Methionine synthase; CBS, cystathionine -β-synthase.
