# Supplementary figures and images for: Hyperhomocysteinemia Increases Risk of Metabolic Syndrome and Cardiovascular Death in an Elderly Chinese Community Population of a 7-Year Follow-Up Study
Source: Front Cardiovasc Med. 2022 Feb 10;8:811670. doi: 10.3389/fcvm.2021.811670 (PMC8870623; doi:10.3389/fcvm.2021.811670)

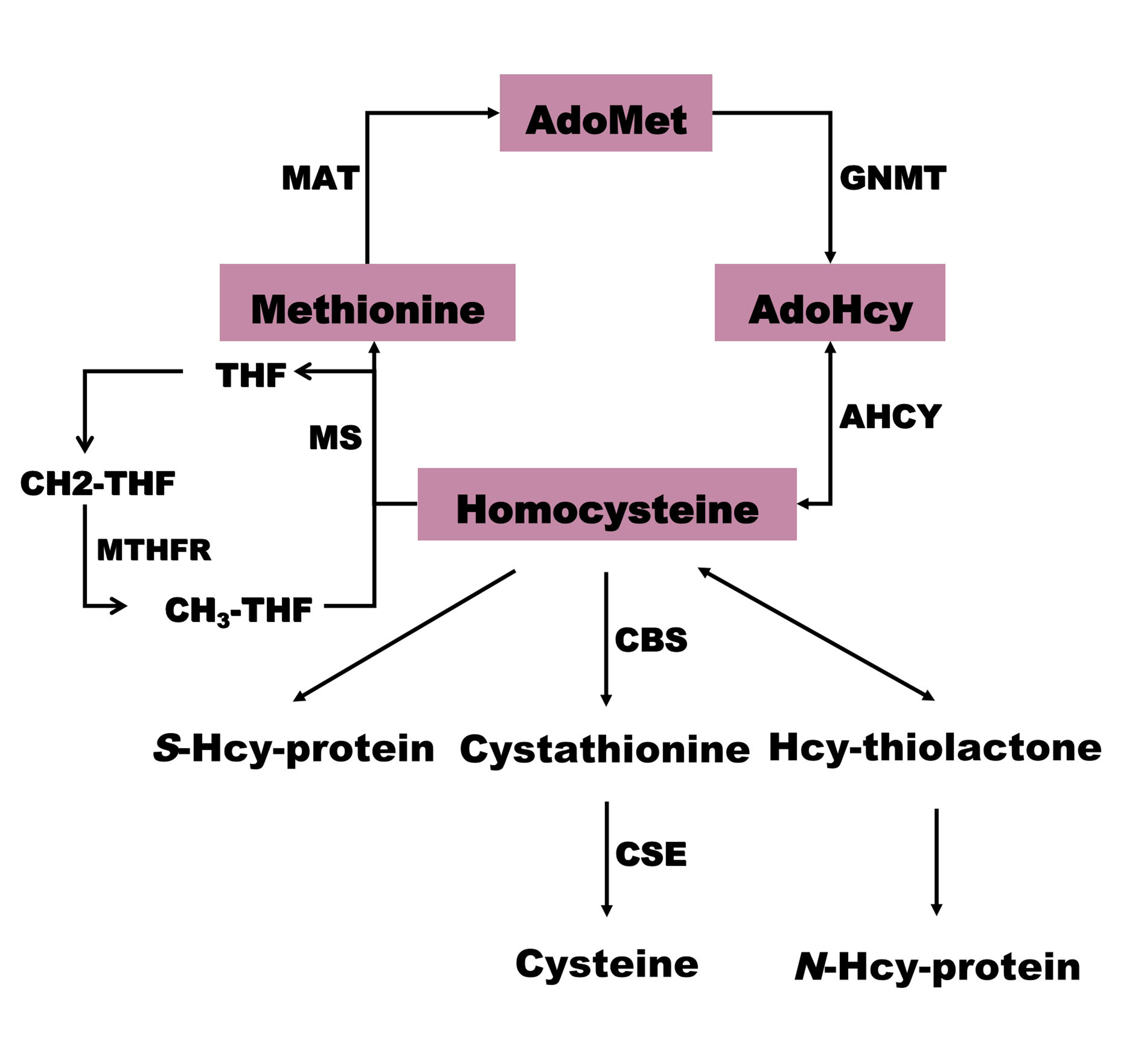

Supplement: Supplementary Figure 1 — The representative figure of homocysteine metabolism in human. AdoMet, adenosylmethionine; MAT, methionine S-adenosyltransferase; GNMT, glycine N-methyltransferase; AdoHcy, S-Adenosyl-L-homocysteine; THF, tetrahydrofolate; MTHFR, methylenetetrahydrofolate reductase; MS, MS, Methionine synthase; CBS, cystathionine -β-synthase. [file Image_1.jpg]
